# Molecular Deregulation of *EPAS1* in the Pathogenesis of Esophageal Squamous Cell Carcinoma

**DOI:** 10.3389/fonc.2020.01534

**Published:** 2020-09-11

**Authors:** Farhadul Islam, Vinod Gopalan, Simon Law, Alfred K. Lam, Suja Pillai

**Affiliations:** ^1^School of Biomedical Sciences, Faculty of Medicine, University of Queensland, Brisbane, QLD, Australia; ^2^Department of Biochemistry and Molecular Biology, University of Rajshahi, Rajshahi, Bangladesh; ^3^School of Medicine, Griffith University, Gold Coast Campus, Gold Coast, QLD, Australia; ^4^Department of Surgery, University of Hong Kong, Hong Kong, China

**Keywords:** ESCC, EPAS1, cancer prognosis, cancer genetics, mutations

## Abstract

Endothelial PAS domain-containing protein 1 (EPAS1) is an angiogenic factor and its implications have been reported in many cancers but not in esophageal squamous cell carcinoma (ESCC). Herein, we aim to examine the genetic and molecular alterations, clinical implications, and functional roles of *EPAS1* in ESCC. High-resolution melt-curve analysis and Sanger sequencing were used to detect mutations in *EPAS1* sequence. *EPAS1* DNA number changes and mRNA expressions were analyzed by polymerase chain reaction. *in vitro* functional assays were used to study the impact of EPAS1 on cellular behaviors. Overall, 7.5% (*n* = 6/80) of patients with ESCC had mutations in *EPAS1*, and eight novel variants (c.1084C>T, c.1099C>A, c.1145_1145delT, c.1093C>G, c.1121T>G, c.1137_1137delG, c.1135_1136insT, and c.1091_1092insT) were detected. Among these mutations, four were frameshift (V382Gfs^*^12, A381Lfs^*^13, K379Ifs^*^6, and K364Nfs^*^12) mutations and showed the potential of non–sense-mediated mRNA decay (NMD) in computational analysis. The majority of patients showed molecular deregulation of *EPAS1* [45% (*n* = 36/80) DNA amplification, 42.5% (*n* = 34/80) DNA deletion, as well as 53.7% (*n* = 43/80) high mRNA expression, 20% (*n* = 16/80) low mRNA expression]. These alterations of *EPAS1* were associated with tumor location and T stages. Patients with stage III ESCC having *EPAS1* DNA amplification had poorer survival rates in comparison to *EPAS1* DNA deletion (*p* = 0.04). In addition, suppression of *EPAS1* in ESCC cells showed reduced proliferation, wound healing, migration, and invasion in comparison to that of control cells. Thus, the molecular and functional studies implied that *EPAS1* plays crucial roles in the pathogenesis of ESCC and has the potential to be used as a prognostic marker and as a therapeutic target.

## Introduction

Hypoxia-inducible factor 1 (HIF1) is an oxygen-sensitive transcription factor consisting of heterodimer of α and β subunits (1). The functional HIF1 is composed of constitutively expressed β subunit and an oxygen-sensitive subunit HIF1α or its isomers HIF2α and HIF3α. These HIF1α isomers are encoded by the *HIF1A, endothelial PAS domain-containing protein 1* (*EPAS1*), and *HIF3A* genes, respectively (2). In hypoxia, HIF1 recognizes the hypoxia response element and regulates the expression of many genes associated with cell proliferation, growth, survival, angiogenesis, and iron and glucose metabolism (1, 3).

HIF2α, an angiogenic factor encoded by *EPAS1* gene, is involved in many physiological and pathological processes, including ferroptosis, endochondral and intramembranous ossification, and Pacak-Zhuang syndrome (4–6). Dysregulation of ferroptosis, a form of regulated cell death, characterized by excessive accumulation of iron and lipid peroxidation, is associated with several diseases such as cancer, neurodegeneration, and ischemia–reperfusion injury (6, 7). Accordingly, it was reported that expression of EPAS1 is associated with pathogenesis, progression, and prognosis of different cancers, including non–small cell lung carcinoma (8), renal cell carcinoma (9), hepatocellular carcinoma (10), neuroblastoma (11), pheochromocytoma (12), glioma (13), and colorectal carcinoma (14). For example, in colorectal carcinoma, EPAS1 protein expression inversely correlated with higher tumor grade and plasma mRNA level of EPAS1 expression and is associated with poor patients' survival and advanced pathological stages (15, 16).

Mutations in the coding sequence of *EPAS1* has been identified in several pathophysiological conditions in human, including congenital heart disease, erythrocytosis, and Lynch syndrome (17–20). In addition, various tumors, e.g., paraganglioma (21), pheochromocytoma (12), and pancreatic adenocarcinoma (22), showed mutations in *EPAS1* sequences. To the best of our knowledge, mutations and their impacts with clinicopathological parameters in patients with ESCC have not been reported in the literature. Also, the molecular deregulations of *EPAS1* and their cellular impact in ESCC have never been studied. Therefore, the present study aims to screen mutations in *EPAS1* sequence in patients with ESCC and their association with clinical and pathological parameters. Furthermore, the EPAS1 DNA number changes, mRNA expression, their correlation with clinical factors, and functional implications of EPAS1 in ESCC cells were investigated in the present study.

## Materials and Methods

### Patients and Clinicopathological Parameters

The clinical samples used in this study were collected from patients who had a surgical resection for primary ESCC. The samples were recruited with no selection bias. Those cancers from patients who underwent preoperative chemoradiotherapy and/or with poor histology were excluded in the present study. Ethic approval was obtained from Griffith University (MED/19/08/HREC) for the present study. The specimens were received fresh after the operation. The age and gender of the patients were noted. In each case, the location and the size of the carcinoma were examined and recorded in fresh. The nonneoplastic esophageal tissues were prospectively collected from the nonneoplastic esophageal mucosa at the proximal resection margin (act as controls) during the operation of the patients with ESCC at the same time of collection of the ESCC tumor tissues. Samples were also collected in 10% buffered formalin and processed in formalin. For each selected sample, tissues were sectioned using a microtome (Leica Biosystems Inc., Buffalo Grove, IL, USA) and stained by hematoxylin–eosin staining for histological analysis by an anatomical pathologist (A.K.L.). The other portion of the resected specimen was fixed in formalin, processed in paraffin, and examined pathologically by the same anatomical pathologist (A.K.L.) using a standard protocol (23). Histological types and grades of selected ESCCs were assessed based on the current World Health Organization histological typing of esophageal tumors prior to analysis (24). Pathological staging was identified according to the current Cancer Staging Manual of the American Joint Committee on Cancer (25).

In this study, 80 patients (67 men, 13 women) with resections of primary ESCC were recruited. In addition, 33 nonneoplastic tissues from esophagus were collected to use as controls. The mean age of the 80 patients with ESCC was 63 years (ranging from 39 to 83 years), and the sizes of the tumors ranged from 5 to 120 mm (mean = 50 mm). The majority of patients (66%, *n* = 53/80) included in this study had stage III ESCCs. In addition, 75% (60/80) of the patients with ESCC had lymph node metastasis at the time of surgery, and 6% (5/80) had distant metastasis at presentation.

**Figure 1:**
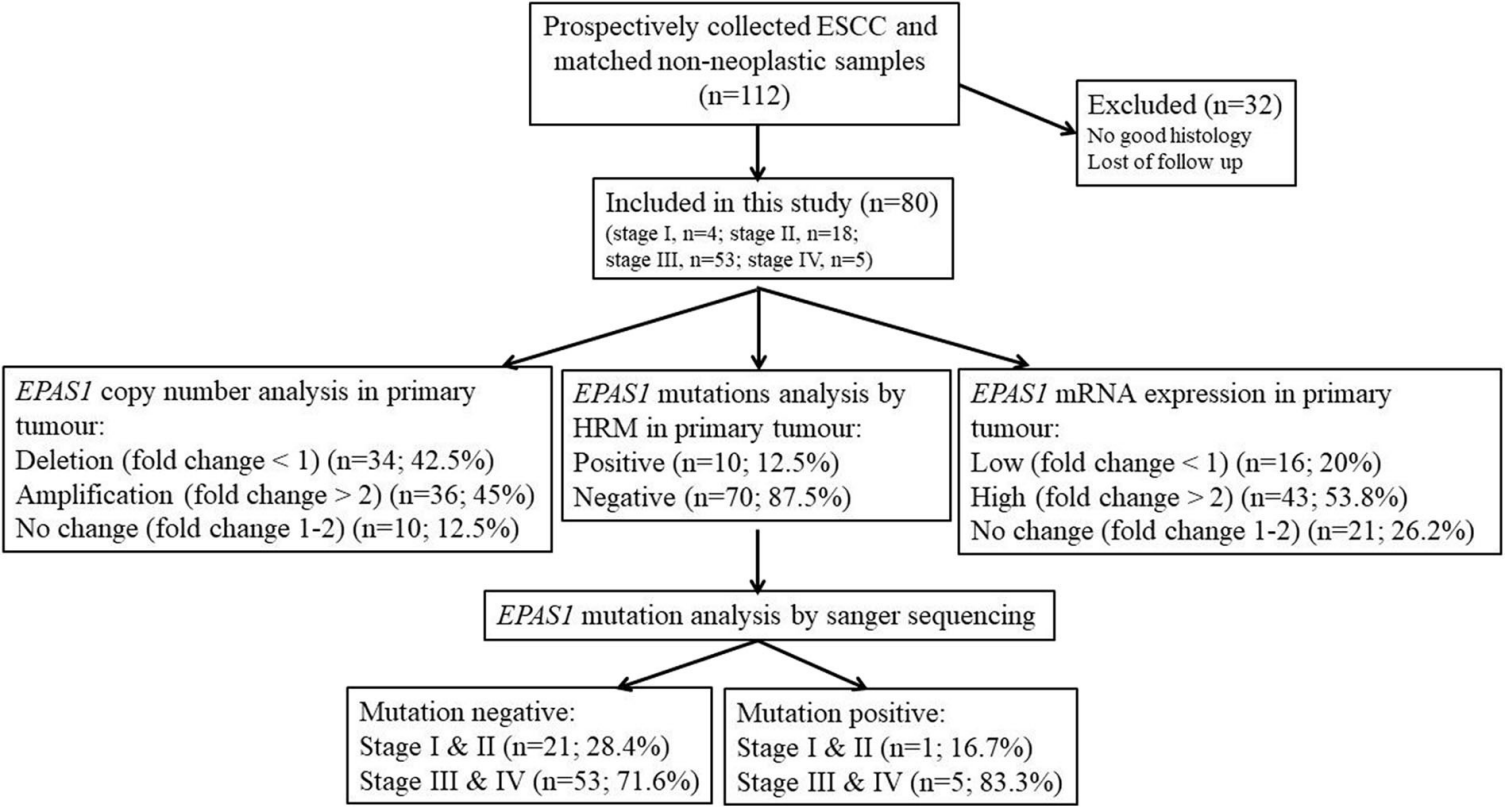
Schematic representation of the methodological flow used for clinical samples analysis in the present study. Tissue samples with poor histology and loss of follow-up were excluded in the present study. Among the samples, 45% showed *EPAS1* DNA amplification, whereas 42.5% showed *EPAS1* DNA deletion. The rest of the samples (12.5%) showed no changes in *EPAS1* DNA. In addition, 53.8% of samples showed high *EPAS1* mRNA expression, and 16.2% of samples showed low *EPAS1* mRNA expression, whereas 20% of samples did not show any changes in *EPAS1* mRNA expression. Furthermore, 12.5% of samples showed *EPAS1* mutations, and 87.5% of samples were mutation negative in the present study.

In this study, the follow-up period was defined as the interval between the date of surgery for ESCC and the date of death or closing date of the study. The actuarial survival rate of the patients was calculated from the date of surgical resection of the ESCC to the date of death or last follow-up. A schematic summary of the flow of the experiments used in the current study is shown in Figure 1.

### Cell Culture

Five ESCC cancer cell lines (KYSE70, KYSE150, KYSE450, KYSE520, and HKESC-1) and one nonneoplastic keratinocyte (HaCaT) were used in the present study. All the cells were maintained as previously described (26, 27).

### Extraction of DNA and RNA

A microtome (Leica Biosystems) was used to section (10 μm) tissues for RNA and DNA extraction. Sections that contained a representative cancer area (made up >70% of the volume of the samples) were used for extraction. DNA was extracted and purified using Qiagen DNeasy Blood & Tissue kits (Qiagen Pty. Ltd., Hilden, Germany) following the manufacturer's guidelines. DNA from cultured cells was extracted with the same kits. In addition, RNA was extracted from the tissue sections and cultured cells using miRNeasy Mini kits (Qiagen) according to the manufacturer's protocol. The purity of the extracted DNA and RNA was checked with optical density using a NanoDrop spectrophotometer. The extracted DNA and RNA were stored at −20°C for further analysis.

### High-Resolution Melt Curve Analysis

Genomic DNAs extracted from 80 cancers and 30 noncancer tissues were used to screen possible mutations in *EPAS1* sequence by high-resolution melt (HRM) analysis. Rotor-Gene Q detection system (Qiagen) was used for amplifying target sequences, followed by HRM curve analyzed using Rotor-Gene ScreenClust HRM Software. The *EPAS1* sequence was amplified via polymerase chain reaction (PCR) in a total reaction volume of 10 μL comprising 5 μL of 2Xsensimix HRM master mix, 1 μL of 30 ng/μL genomic DNA, diethylpyrocarbonate (DEPC, RNase-free) treated water 2 and 1 μL of each forward and reverse *EPAS1* primer. The thermal cycling protocol was the same as published previously (28). The melt curve data were generated by increasing the temperature from 65 to 85°C for all assays, with a temperature increase rate of 0.05°C/s and recording fluorescence. All the samples were run in triplicates and included a negative (no template) control.

### Purification of PCR Products and Sanger Sequencing Analysis

The variants detected in HRM analysis were further confirmed via checking with Sanger sequencing for identifying the mutations in *EPAS1* sequence. Briefly, after HRM analysis, PCR products from mutant samples were purified using NucleoSpin® Gel and PCR Clean-up kit (Macherey- Nagel, Bethlehem, PA, USA) according to the manufacturer's protocols. Then, the purified PCR products were sequenced using Big Dye Terminator Chemistry version 3.1 (Applied Biosystems, Foster City, CA, USA) under standardized cycling PCR conditions. The generated data were analyzed at the Australian Genome Research Facility using a 3730xl Capillary sequencer (Applied Biosystems). The sequences were analyzed with Sequence Scanner 2 software (Applied Biosystems).

### *In silico* Analysis

The Ensembl transcript ID ENST00000263734 was used as input when required by a method. In this study, all the variants were analyzed using freely available bioinformatics tools such as Mutation Taster with NCBI 37 and Ensembl 69 database release (29), PROVEAN (protein variation effect analyzer), and SIFT (sorting intolerant from tolerant) to evaluate the consequences of the identified mutations. In addition, results were compared with ExAc and 1000 Genomes variant databases to check the single-nucleotide polymorphism. In the current study, the cutoff value for PROVEAN and SIFT analysis was used as −2.5 and 0.05, respectively, for predicting the pathogenic/nonpathogenic variants.

### Quantitative Real-Time PCR (qPCR) Analysis

DNA copy number changes of *EPAS1* in ESCC (*n* = 80) and noncancerous (*n* = 30) tissues were examined using QuantStudio 6 Flex Real-Time PCR System (Thermo Fisher Scientific, Waltham, MA, USA). Briefly, quantitative PCR (qPCR) was performed in a total volume of 20 μL reaction mixture containing 10 μL of DyNAmo Flash SYBR Green Master Mix (Bio-Rad, Gladesville, New South Wales, Australia), 1.5 μL of each 5 μmol/L forward and reverse primer, 3 μL of DNA at 50 ng/μL, and 4 μL of 0.1% DEPC-treated water as previously described (30).

For qPCR, first-strand cDNA was generated using DyNAmo™ cDNA Synthesis Kits (Qiagen) as previously described (31). EPAS1 mRNA expression changes in ESCC samples were examined using QuantStudio 6 Flex Real-Time PCR System (Thermo Fisher Scientific). In short, qPCR was performed in a total volume of 20 μL reaction mixture containing 10 μL of DyNAmo Flash SYBR Green Master Mix (Bio-Rad), 1.5 μL of each 5 μmol/L forward and reverse primer, 1 μL of cDNA at 50 ng/μL, and 4 μL of 0.1% DEPC-treated water as previously described (30). The amplification efficiencies were normalized to that of multiple housekeeping genes, including β-actin, 18s, and glyceraldehyde 3-phosphate dehydrogenase (GAPDH). GAPDH and β-actin were selected based on consistent results. Results were presented as a ratio of expression (expression of *EPAS1* normalized by internal control *GAPDH* and β*-actin* expression) in ESCC tissue samples and cells. Fold changes were calculated according to a previously published protocol (32), and a fold change of more than 2 was considered as high *EPAS1* expression or amplification, a fold change of 1.0–2.0 was considered as no change, and a fold change of <1.0 was considered as low *EPAS1* expression or deletion.

### Transfection of ESCC Cells With *EPAS1* siRNA Silencer and Scramble siRNA

KYSE70 and KYSE150 ESCC cells were seeded approximately at 2 × 10^4^ cells/cm^2^ into 24-well plate in the growth media (26). After 24 h of initial seeding, cells were transfected with *EPAS1* siRNA silencer (Qiagen) (KYSE70^−EPAS1^ and KYSE150^−EPAS1^) at 15-nM concentrations and with scramble siRNA (Qiagen) (KYSE70^+Scr^ and KYSE150^+Scr^) at 10-nM concentrations according to the manufacturer's guidelines. Briefly, 3 μL of the transfection reagent, Hiperfect (Qiagen), was added to the siRNAs and incubate for 5 min at room temperature to form the complexes. Then, cells were treated with the complex and used for functional assays. Cells treated with scrambled siRNA (KYSE70^+Scr^ and KYSE150^+Scr^) and transfection reagents (Hiperfect) alone (KYSE70^wildtype^ and KYSE150^wildtype^) were used as controls in the present study.

### Western Blot Analysis

Total proteins were extracted from the cultured cells with lysis buffer (Bio-Rad) and quantitation by bovine serum albumin method. Afterward, total protein (30 μg) was separated by 15% sodium dodecyl sulfate–polyacrylamide gel electrophoresis (Bio-Rad) and transferred to polyvinylidene fluoride membranes (Bio-Rad) using Turbo Trans-blot transfer system (Bio-Rad). Then, the membrane was incubated with mouse monoclonal EPAS1 and GAPDH antibody (1:1,000) at 4°C overnight with gentle shaking. The membrane was then incubated with anti–mouse secondary antibody (1:2,000) at room temperature for 2 h. Finally, the blots were developed to detect protein bands according to the published protocol (33).

### Cell Proliferation Assay

To examine the effect of EPAS1 on the proliferation of ESCC, cell proliferation assay was performed using cell counting kit-8 (CCK-8) (Sigma-Aldrich, St Louis, MO, USA) (34). Briefly, KYSE70 and KYSE150 cells were seeded in a flat-bottom 96-well plate at 1 × 104 cells/well. After 24 h of initial seeding, cells were treated with EPAS1 siRNA silencer and scramble siRNA as previously described (34). Then, the proliferation rate of EPAS1 siRNA-treated and controls cells was determined on days 0 to 3 with CCK-8 following manufacturer guidelines.

### Colony Formation Assay

To determine the effect of EPAS1 manipulation on clonogenic capacity of ESCC, equal numbers (~1,000) of cells (KYSE70 and KYSE150) were seeded in six-well plates and were then transfected with EPAS1 siRNA and scramble siRNA. Cells were grown (for 14–16 days) at 37°C in 5% carbon dioxide and saturation humidity until microscopic clones were noted in the plate. After that, the media was discarded, and cells were washed with a phosphate-buffered saline solution. The cells were then fixed with 70% cold ethanol for 15 min at room temperature. Subsequently, the clones were stained with crystal violet (0.5%) for 2 h at room temperature and washed with tap water. Finally, after being air-dried, images of the plates were taken, and clone formation rates were calculated as previously described (26).

### Wound Healing Assay

To examine the effect of EPAS1 on the capacity of cells of ESCC to migrate for repairing, the scratch wound healing assay were used as previously reported (35). In short, KYSE70 and KYSE150 cells were grown in the medium until 70–80% confluence as a monolayer, and scratches were made using a 200-μL pipette tip across the center of culture plates. The cells were later treated with EPAS1 siRNA and scramble siRNA (control siRNA) and incubated for analysis of the migration of cells to heal the wound. Images were taken to monitor the changes among the cells type on days 0 to 2, and wound areas on different days of all cell types were recorded.

### Invasion Assay

To investigate the silencing effect of EPAS1 on ESCC cells' *in vitro* cell penetration/invasion to a barrier, CultreCoat® 96-well basement membrane extract (BME)–coated cell invasion assay (Trevigen Inc., Gaithersburg, MD, USA) kit with basement membrane components was used following the protocol previously published (36). In brief, KYSE70 and KYSE150 cells were cultured to 80% confluence and passaged to a serum-free medium for 24 h. Then, the serum-starved cells were collected, and 50 μL (1 × 10^6^/mL) of cell suspension was added to each well of 96-well top chamber. After that, the transfection complex consisting of EPAS1 siRNA and Hiperfect transfection reagent (Qiagen) was added to the top chamber to transfect the cells. Similarly, scramble siRNA and transfection reagent (Hiperfect) was added in wells to be used as control. Then, the complete growth media was added to the bottom chamber of the assay kit and incubated at 37°C in 5% carbon dioxide incubator for 48 h. After incubation, 100-μL cell dissociation solution/calcein AM was added to the bottom chamber, which allows internalization of calcein AM to the cells, and intracellular esterase cleaves it to produce calcein (a bright fluorophore). Finally, the fluorescence generated by the invaded cells was used to quantitate the number of invasive cells in each group with POLARstar Omega multimode microplate reader (BMGLABTECH, Mornington, Victoria, Australia).

### Statistical Analysis

Comparisons between variable groups were analyzed using the χ^2^ test, likelihood ratio, and Fisher exact test. All the data were entered into a computer database, and the statistical analysis was executed using the Statistical Package for Social Sciences for Windows (version 25.0; IBM SPSS Inc., New York, NY, USA). Survival analysis was tested using Kaplan–Meier method. Results are shown as mean ± SD (standard deviation), and the significance level was taken at *p* < 0.05. ^*^p < 0.05, ^**^p < 0.01, and ^***^p < 0.001.

## Results

### Identification of Novel *EPAS1* Mutations in ESCC Tissue Samples

**Figure 2:**
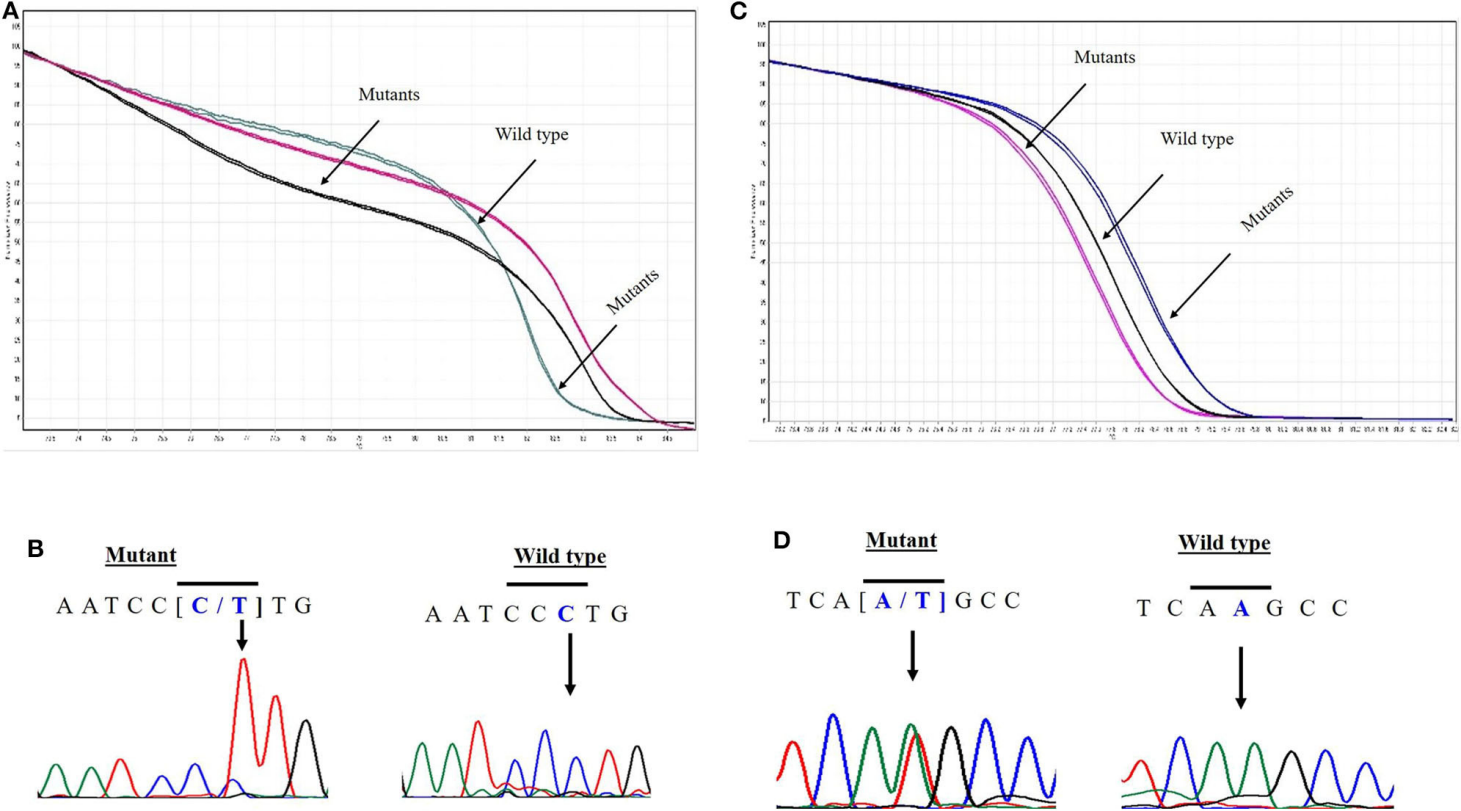
Novel variants in *EPAS1* detected in ESCC tissues. Comparison of HRM curve analysis and Sanger sequencing of the variants identified in patients with ESCC. Representative HRM curve **(A)** and chromatograph **(B)** for the synonymous mutation c.1084C>T (L362L). Representative HRM curve **(C)** and chromatograph **(D)** for the frameshift variant c.1091_1092insT (K364Nfs*12).

*EPAS1* mutant variants were detected in tissues based on the distinctive melting curve of HRM analysis and then confirmed with Sanger sequencing (Figure 2). In the present study, 7.5% (*n* = 6) of 80 patients had mutations in *EPAS1* sequence. There were eight variants (c.1084C>T, c.1099C>A, c.1145_1145delT, c.1093C>G, c.1121T>G, c.1137_1137delG, c.1135_1136insT, and c.1091_1092insT) identified in the coding region of *EPAS1* (Table 1). Among these mutations, four were frameshift (V382Gfs^*^12, A381Lfs^*^13, K379Ifs^*^6, and K364Nfs^*^12) mutations. No mutant variant was detected in noncancerous control tissues.

**Table 1:** Mutations detected in the sequence of *EPAS1* in esophageal squamous cell carcinoma.

**Sample** **ID**	**Copy No.** **Change**	**mRNA** **expression**	**DNA** **change**	**Amino acid changes**	**Effect on protein features**	***In silico*** **prediction**		
						**Mutation taster**	***PROVEAN***	***SIFT***
P1	Amplification	High	c.1084C>T cDNA.1594C>T g.82922C>T	No	Protein features (might be) affected	Diseases causing	Neutral	Tolerated
P13	Amplification	High	c.1099C>A cDNA.1609C>A g.82937C>A c.1145_1145delT cDNA.1655_1655delT g.82983_82983delT	L367M V382Gfs^*^12	Amino acid sequence changed NMD Amino acid sequence changed Frameshift protein features (might be) affected	Diseases causing	Neutral Deleterious	Tolerated Deleterious
P22	Deletion	No change	c.1093C>G cDNA.1603C>G g.82931C>G c.1099C>A cDNA.1609C>A g.82937C>A c.1145_1145delT cDNA.1655_1655delT g.82983_82983delT	P365A L367M V382Gfs^*^12	Amino acid sequence changed Amino acid sequence changed NMD Amino acid sequence changed Frameshift protein features (might be) affected	Diseases causing	Deleterious	Damaging
P29	Amplification	High	c.1099C>A cDNA.1609C>A g.82937C>A c.1121T>G cDNA.1631T>G g.82959T>G c.1137_1137delG cDNA.1647_1647delG g.82975_82975delG	L367M F374C A381Lfs^*^13	Amino acid sequence changed Amino acid sequence changed NMD amino acid sequence changed frameshift protein features (might be) affected splice site changes	Diseases causing	Deleterious	Damaging
P78	Amplification	High	c.1135_1136insT cDNA.1645_1646insT g.82973_82974insT c.1099C>A cDNA.1609C>A g.82937C>A	K379Ifs^*^6 L367M	NMD Amino acid sequence changed Frameshift Protein features (might be) affected Splice site changes Amino acid sequence changed	Diseases causing	Deleterious	Damaging
P103	Deletion	Low	c.1091_1092insT cDNA.1601_1602insT g.82929_82930insT	K364Nfs^*^12	NMD Amino acid sequence changed Frameshift Protein features (might be) affected Splice site changes	Diseases causing	Deleterious	Damaging

*NMD, nonsense-mediated mRNA decay*.

The consequences of nucleotides, as well as amino acid changes on protein features and functions, were predicted by computational analysis (Table 1). All the variants identified in the present study in *EPAS1* were predicted as deleterious or damaging on the functionality of EPAS1 protein in ESCC (Table 1). In addition, the detected variants are novel as the identified variants were not found in the ExAc and 1000 Genomes variant databases or in the PubMed database.

The associations of the *EPAS1* mutations with clinicopathological factors are summarized in Table 2. Clinicopathological factors such as site, size, differentiation, and pathological stages were not associated with *EPAS1* mutations. Mutations in *EPAS1* sequence correlated with patient's age (*p* = 0.02) and the presence of metastatic carcinoma in lymph node (*p* = 0.05). Overall, 10% (*n* = 6/60) of ESCCs with metastatic carcinoma in the lymph node had *EPAS1* mutations, whereas no mutation was detected in ESCC without lymph node metastasis.

**Table 2:** Correlation of *EPAS1* mutations with clinicopathological features of patients with esophageal squamous cell carcinoma.

**Features**	**Number**	**Negative**	**Positive**	**P-value**
**Total patients**		80	74 (92.5%)	6 (7.5%)
**Sex**				
Male	67 (83.8%)	62 (92.5%)	5 (7.5%)	0.66
Female	13 (16.2%)	12 (92.3%)	1 (7.7%)	
**Age**				
≤60	54 (67.5%)	48 (88.9%)	6 (11.1%)	**0.02**
>60	26 (32.5%)	26 (100%)	0 (0%)	
**Site**				
Upper or middle	53 (66.3%)	50 (94.3%)	3 (5.7%)	0.32
Lower	27 (33.7%)	24 (88.9 %)	3 (11.1%)	
**Size (cm)**				
≤6	31 (38.7%)	29 (93.5%)	2 (6.5%)	0.57
>6	49 (61.3%)	45 (91.8%)	4 (8.2%)	
**Differentiation**				
Well	24 (30.0%)	23 (95.8%)	1 (4.2%)	0.65
Moderate	39 (48.8%)	36 (93.3%)	3 (7.7%)	
Poor	17 (21.2%)	15 (88.2%)	2 (11.8%)	
**T-stages**				
*I & II*	6 (7.5%)	5 (83.3%)	1 (16.7%)	0.38
*III & IV*	74 (92.5%)	69 (93.2%)	5 (6.8%)	
**Lymph-node metastasis**				
Presence	60 (75.0%)	54 (90.0%)	6 (10.0%)	**0.05**
Absence	20 (25.0%)	20 (100%)	0 (0.0%)	
**Distant metastasis**				
Yes	5 (6.3%)	4 (80.0%)	1 (20.0%)	0.33
No	75 (93.7%)	70 (93.3%)	5 (6.7%)	
**Stage**				
*I & II*	22 (27.5%)	21 (95.5%)	1 (4.5%)	0.47
*III & IV*	58 (72.5%)	53 (91.4%)	5 (8.6%)	

*Bold values indicates p-value of 0.05 or below*.

### *EPAS1* DNA Changes and mRNA Deregulation in ESCC

**Figure 3:**
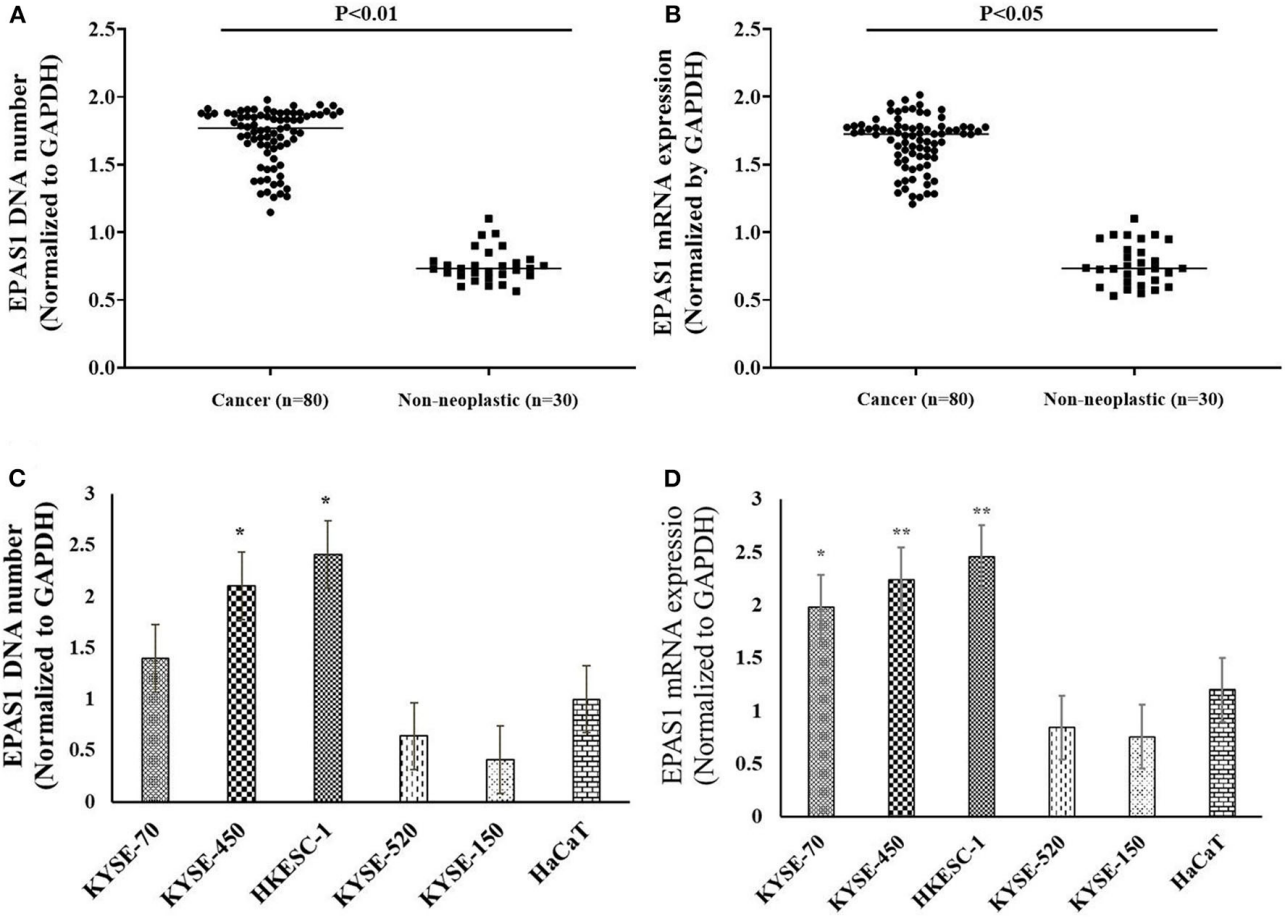
*EPAS1* DNA number and mRNA expression profile in patients with ESCC and cell lines. **(A)** Patients with ESCC exhibited significant amplifications of *EPAS1* DNA when compared with noncancerous samples (*p* < 0.01). **(B)** Similarly, a significant overexpression of *EPAS1* mRNA in ESCC was noted in comparison to that of noncancerous tissues (*p* < 0.05). **(C)** Cell lines showed higher or lower *EPAS1* DNA number when compared to that of noncancerous keratinocyte (HaCaT) cells. **(D)** Higher or lower *EPAS1* mRNA was noted in ESCC cancer cells when compared with nonneoplastic HaCaT cells. Results are shown as mean ± SD, and significance level was taken at *p* < 0.05. *p < 0.05, and **p < 0.01.

In the present study, 45% (*n* = 36) of the 80 ESCC samples showed *EPAS1* DNA amplification, whereas 42.5% (*n* = 34) showed deletion in comparison to the noncancer tissue samples (Table 3). The rest of the samples (12.5%; *n* = 10) did not exhibit any changes in *EPAS1* DNA copies (Table 3). The distribution of *EPAS1* DNA in cancer and noncancer tissue samples is shown in Figure 3A. A significantly higher *EPAS1* DNA expression was noted in cancer samples (1.706 ± 0.209) when compared with noncancerous (0.569 ± 0.078) samples.

**Table 3:** Correlation of *EPAS1* DNA variations with clinicopathological features of patients with esophageal squamous cell carcinoma.

**Features**	**Number**	**Amplification**	**Deletion**	**No change**	**P-value**
**Total patients**	80	36 (45.0%)	34 (42.5%)	10 (12.5%)	–
**Sex**					
Male	67 (83.8%)	33 (49.3%)	26 (38.8%)	8 (11.9%)	0.19
Female	13 (16.2%)	3 (23.1%)	8 (61.5%)	2 (15.4%)	
**Age**					
≤60	54 (67.5%)	22 (40.7%)	25 (46.3%)	7 (13.0%)	0.53
>60	26 (32.5%)	14 (53.9%)	9 (34.6%)	3 (11.5%)	
**Site**					
Upper or middle	53 (66.3%)	19 (35.8%)	25 (47.2%)	9 (17.0%)	**0.03**
Lower	27 (33.7%)	17 (63.0 %)	9 (33.3%)	1 (3.7%)	
**Size (cm)**					
≤6	31 (38.7%)	12 (38.7%)	12 (38.7%)	7 (22.6%)	0.09
>6	49 (61.3%)	24 (49.0%)	22 (44.9%)	3 (6.1%)	
**Differentiation**					
Well	24 (30.0%)	12 (50.0%)	9 (37.5%)	3 (12.5%)	0.89
Moderate	39 (48.8%)	18 (46.2%)	16 (41.0%)	5 (12.8%)	
Poor	17 (21.2%)	6 (35.2%)	9 (53.0%)	2 (11.8%)	
**T-stages**					
*I*	3 (3.8%)	2 (66.7%)	1 (33.3%)	–	**0.02**
*II*	3 (3.8%)	1 (33.3%)	2 (66.7%)	–	
*III*	53 (66.2%)	21 (39.6%)	28 (52.8%)	4 (7.6%)	
*IV*	21 (26.2%)	12 (57.1%)	3 (14.3%)	6 (28.6%)	
**Lymph-node metastasis**					
Presence	60 (75.0%)	29 (48.3%)	22 (36.7%)	9 (15.0%)	0.14
Absence	20 (25.0%)	7 (35.0%)	12 (60.0%)	1 (5.0%)	
**Distant metastasis**					
Yes	5 (6.3%)	2 (40.0%)	3 (60.0%)	-	0.43
No	75 (93.7%)	34 (45.3%)	31 (41.3%)	10 (13.4%)	
**Stage**					
*I & II*	22 (27.5%)	8 (36.4%)	13 (59.1%)	1 (4.5%)	0.12
*III & IV*	58 (72.5%)	28 (48.3%)	21 (36.2%)	9 (15.5%)	

*Bold values indicates p-value of 0.05 or below*.

The associations of *EPAS1* DNA changes with clinicopathological parameters of the patients with ESCC are presented in Table 3. We observed that *EPAS1* DNA amplification significantly (*p* < 0.05) correlated with the tumor site and pathological stages in patients with ESCC. ESCCs located at the lower portion of the esophagus had significantly more *EPAS1* DNA amplification in comparison to those from the upper or middle part of the esophagus (63.0 vs. 35.8%; *p* = 0.03). Higher frequency of patients with ESCC having tumor stage I and IV showed *EPAS1* DNA amplification, whereas the majority of the patients with ESCC having tumor stages II and III showed *EPAS1* DNA deletion (*p* = 0.02).

The expressions of *EPAS1* mRNA in cancer and nonneoplastic tissue samples were presented in Figure 3B. The distribution of *EPAS1* mRNA expression in cancer tissues was significantly (1.656 ± 0.193 vs. 0.573 ± 0.078; *p* < 0.05) higher when compared with nonneoplastic tissue samples (Figure 3B). In addition, the mRNA expression ratio of *EPAS1* was significantly higher in cancer in comparison to those in noncancer tissue samples (1.656 ± 0.12 vs. 0.573 ± 0.07; *p* < 0.001). Among the patients' samples used in this study, 53.7% (*n* = 43/80) had higher *EPAS1* mRNA expression, whereas the remaining 20% (*n* = 16/80) exhibited *EPAS1* mRNA lower expression. The rest of the samples (*n* = 21/80; 26.3%) had no changes in *EPAS1* mRNA expression (Table 4). The association of *EPAS1* mRNA expression and the clinicopathological parameters of patients with ESCC were analyzed (Table 4). It was noted that *EPAS1* mRNA expression was not associated with the clinical–pathological parameters of patients with ESCC (Table 4; *p* > 0.05).

**Table 4:** Correlation of *EPAS1* mRNA expression with clinicopathological features of patients with esophageal squamous cell carcinoma.

**Features**	**Number**	**High**	**Low**	**No change**	**P-value**
**Total patients**	80	43 (53.7%)	16 (20.0%)	21 (26.3%)	–
**Sex**					
Male	67 (83.8%)	39 (58.2%)	13 (19.4%)	15 (22.4%)	0.14
Female	13 (16.2%)	4 (30.8%)	3 (23.1%)	6 (46.1%)	
**Age**					
≤60	54 (67.5%)	31 (57.4%)	10 (18.5%)	13 (24.1%)	0.63
>60	26 (32.5%)	12 (46.2%)	6 (23.1%)	8 (30.7%)	
**Site**					
Upper or middle	53 (66.3%)	26 (49.1%)	12 (22.6%)	15 (28.3%)	0.48
Lower	27 (33.7%)	17 (63.0 %)	4 (14.8%)	6 (22.2%)	
**Size (cm)**					
≤6	31 (38.7%)	13 (41.9%)	7 (22.6%)	11 (35.5%)	0.21
>6	49 (61.3%)	30 (61.2%)	9 (18.4%)	10 (20.4%)	
**Differentiation**					
Well	24 (30.0%)	15 (62.5%)	4 (16.7%)	5 (20.8%)	0.75
Moderate	39 (48.8%)	21 (53.8%)	8 (20.5%)	10 (25.7%)	
Poor	17 (21.2%)	7 (41.2%)	4 (23.5%)	6 (35.3%)	
**T-stages**					
*I & II*	6 (7.5%)	2 (33.3%)	1 (16.7%)	3 (50.0%)	0.38
*III & IV*	74 (92.5%)	41 (55.4%)	15 (20.3%)	18 (24.3%)	
**Lymph-node metastasis**					
Presence	60 (75.0%)	34 (56.6%)	13 (21.7%)	13 (21.7%)	0.26
Absence	20 (25.0%)	9 (45.0%)	3 (15.0%)	8 (40.0%)	
**Distant metastasis**					
Yes	5 (6.3%)	2 (40.0%)	1 (20.0%)	2 (40.0%)	0.75
No	75 (93.7%)	41 (54.7%)	15 (20.0%)	19 (25.3%)	
**Stage**					
*I & II*	22 (27.5%)	11 (50.0%)	3 (13.6%)	8 (36.4%)	0.39
*III & IV*	58 (72.5%)	32 (55.2%)	13 (22.4%)	13 (22.4%)	

The number of *EPAS1* DNA in cancer cells is presented in Figure 3C. *EPAS1* DNA numbers (1.4 ± 0.07, 2.10 ± 0.10, 2.41 ± 0.12) in ESCC cancer cell lines KYSE70, KYSE450 and HKESC-1, respectively, are higher when compared with that of nonneoplastic keratinocyte HaCaT (1.01 ± 0.05) cells (Figure 3C). Similarly, the mRNA expression of *EPAS1* cancer cells (KYSE70, KYSE450, and HKESC-1) is significantly higher (1.98 ± 0.09, 2.24 ± 0.11, 2.45 ± 0.12, respectively) than noncancerous HaCaT (1.2 ± 0.06) cells (Figure 3D). However, KYSE520 and KYSE150 did not show any significant difference in *EPAS1* DNA number and mRNA expression when compared with nonneoplastic keratinocyte HaCaT cells (Figures 3C,D).

### Association of *EPAS1* Molecular Deregulation With Patient's Survival

**Figure 4:**
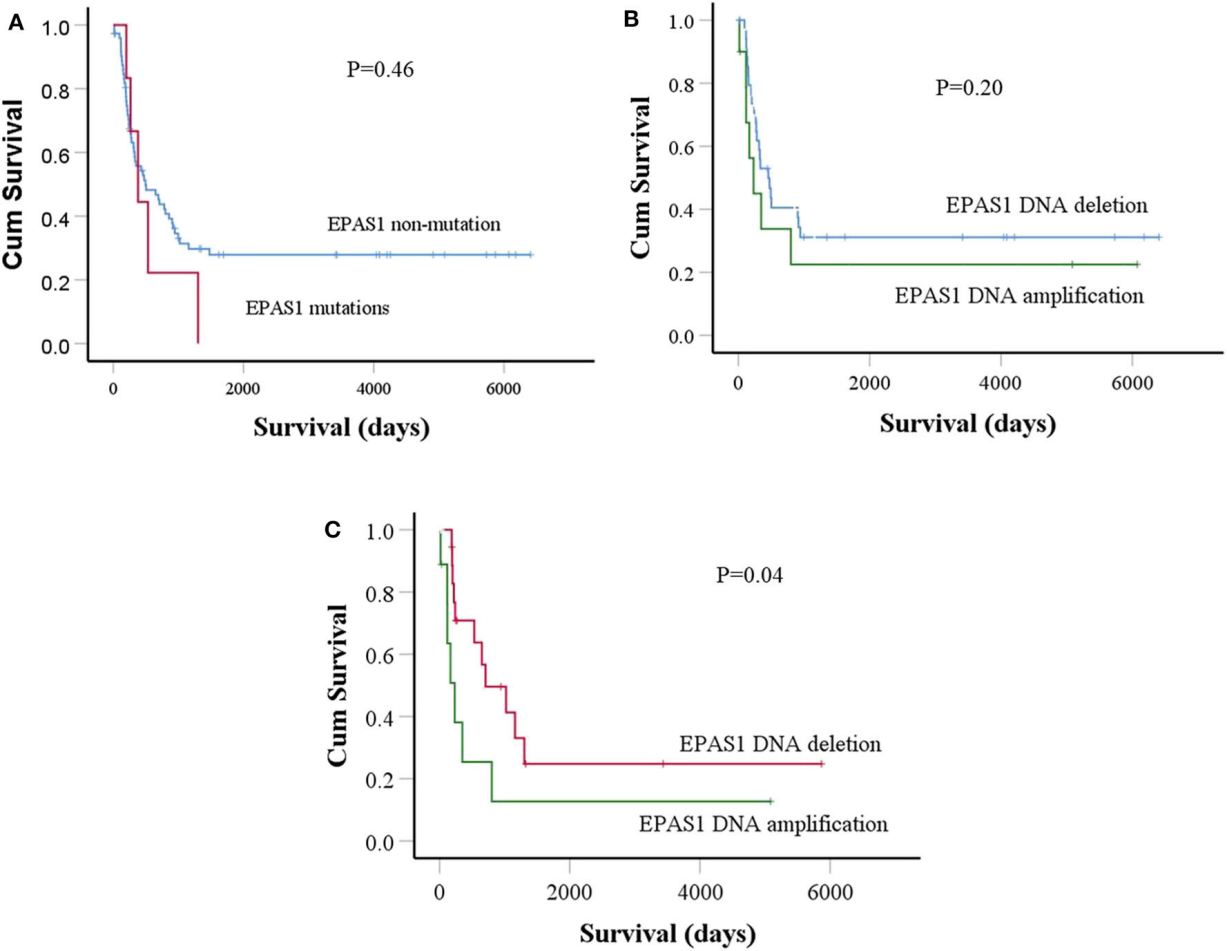
Prognostic significance of *EPAS1* dysregulation in ESCC. **(A)** The trends of *EPAS1*-mutated positive patients had shorter survival rates compared to the nonmutated patients. However, the difference did not reach statistical significance level (*p* = 0.46). **(B)** Patients with *EPAS1* DNA amplification had poorer survival than *EPAS1* DNA deletion (*p* = 0.20). **(C)** In stage III patients with ESCC, the survival rates of patients having *EPAS1* DNA amplification is significantly poor when compared to that of *EPAS1* DNA deletion (*p* = 0.04).

Finally, the prognostic significance of *EPAS1* in patients with ESCC was analyzed. The median overall follow-up of patients with ESCC used in this study was 60 months and the survival rates correlated with the pathological stages of cancer (*p* = 0.0001). Patients with ESCCs harboring mutations in *EPAS1* sequence have poorer survival rates than the patients without *EPAS1* mutations (570.89 ± 205.02 vs. 2,097.15 ± 332.09 days; *p* = 0.46) (Figure 4A). Patients with ESCC having *EPAS1* DNA number amplification showed short survival when compared with that of *EPAS1* DNA deletion (1,568.62 ± 515.31 vs. 2,239.18 ± 489.48 days; *p* = 0.2), although the difference in survival time between the groups did not reach statistical significance (Figure 4B). On the other hand, the survival of patients with stage III ESCC having *EPAS1* DNA amplification showed a significant reduction in patient survival compared to those of stages III patients with *EPAS1* DNA deletion (873.79 ± 576.85 vs. 1,936.63 ± 622.19 days, *p* = 0.04) (Figure 4C).

### Association of *EPAS1* Mutations, DNA Alteration, and mRNA Expression in Patients With ESCC

**Figure 5:**
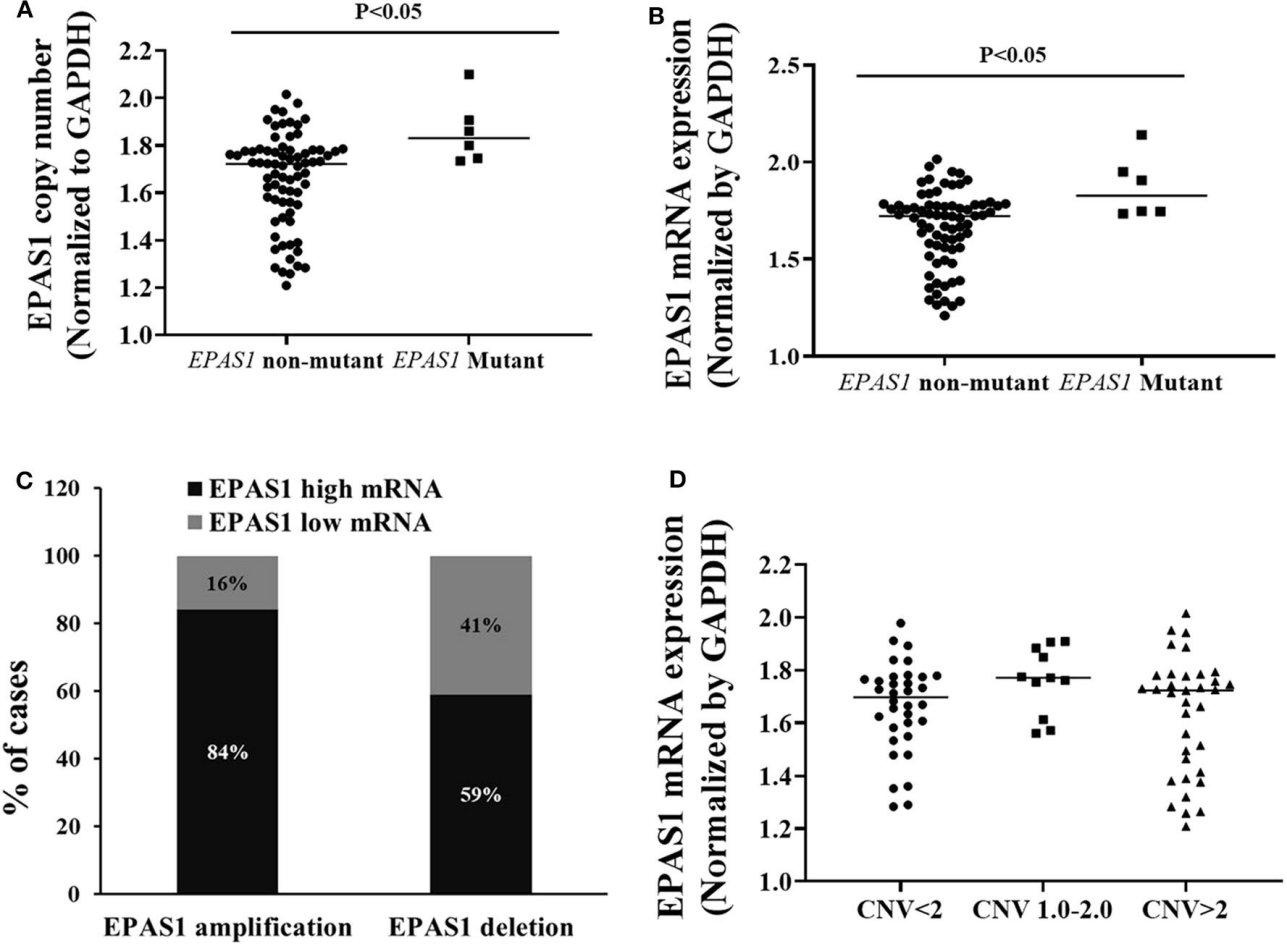
Relationship of *EPAS1* molecular dysregulation in ESCC. **(A)** *EPAS1*-mutated samples showed significant amplification of DNA number in comparison to that of nonmutated samples (*p* < 0.05). **(B)** Similarly, *EPAS1*-mutated samples exhibited significant higher expression (mRNA) when compared to that of nonmutated tissue samples (*p* < 0.05). **(C)** Association of *EPAS1* DNA number changes and mRNA expression. RT-qPCR analysis revealed that *EPAS1* DNA number amplification significantly correlated with mRNA overexpression (*p* < 0.01). A 84% patients having *EPAS1* DNA amplification showed mRNA overexpression whereas 59% patients with *EPAS1* DNA deletion showed mRNA overexpression. **(D)** Distribution of *EPAS1* mRNA expression vs. *EPAS1* DNA number in patients with ESCC. Patients with DNA number greater 2 showed higher mRNA expression and DNA number <2 showed lower *EPAS1* mRNA expression.

The relationships of *EPAS1* mutations, DNA number, and mRNA expression in patients with ESCC were analyzed (Figure 5). ESCCs bearing *EPAS1* mutations showed significantly higher DNA number (1.736 ± 0.241 vs. 1.701 ± 0.204) in comparison to those without the mutation (Figure 5A). Similarly, ESCC with *EPAS1* mutations exhibited significant overexpression (1.741 ± 0.084 vs. 1.564 ± 0.192) of *EPAS1* mRNA level when compared with those without the mutation (Figure 5B).

A statistically significant positive correlation was noted between *EPAS1* DNA number amplification and mRNA overexpression (*r* = 0.468; *p* = 0.01, Fisher exact test). In addition, 84% (30/36) of ESCCs having *EPAS1* DNA amplification had overexpression of *EPAS1* mRNA level. Similarly, *EPAS1* mRNA downregulation was noted in 59% (*n* = 20) of the 34 ESCCs with *EPAS1* DNA deletion (Figure 5C). Moreover, *EPAS1* mRNA expression changes notably with the changes of *EPAS1* DNA variations in ESCC (Figure 5D). In addition, The *EPAS1* mRNA expression changes were also correlated with *EPAS1* DNA copy number variations in ESCC (*p* = 0.05).

### Suppression of *EPAS1* Decreases the Proliferation and Colony Formation Capacity of Colon Cancer Cells

**Figure 6:**
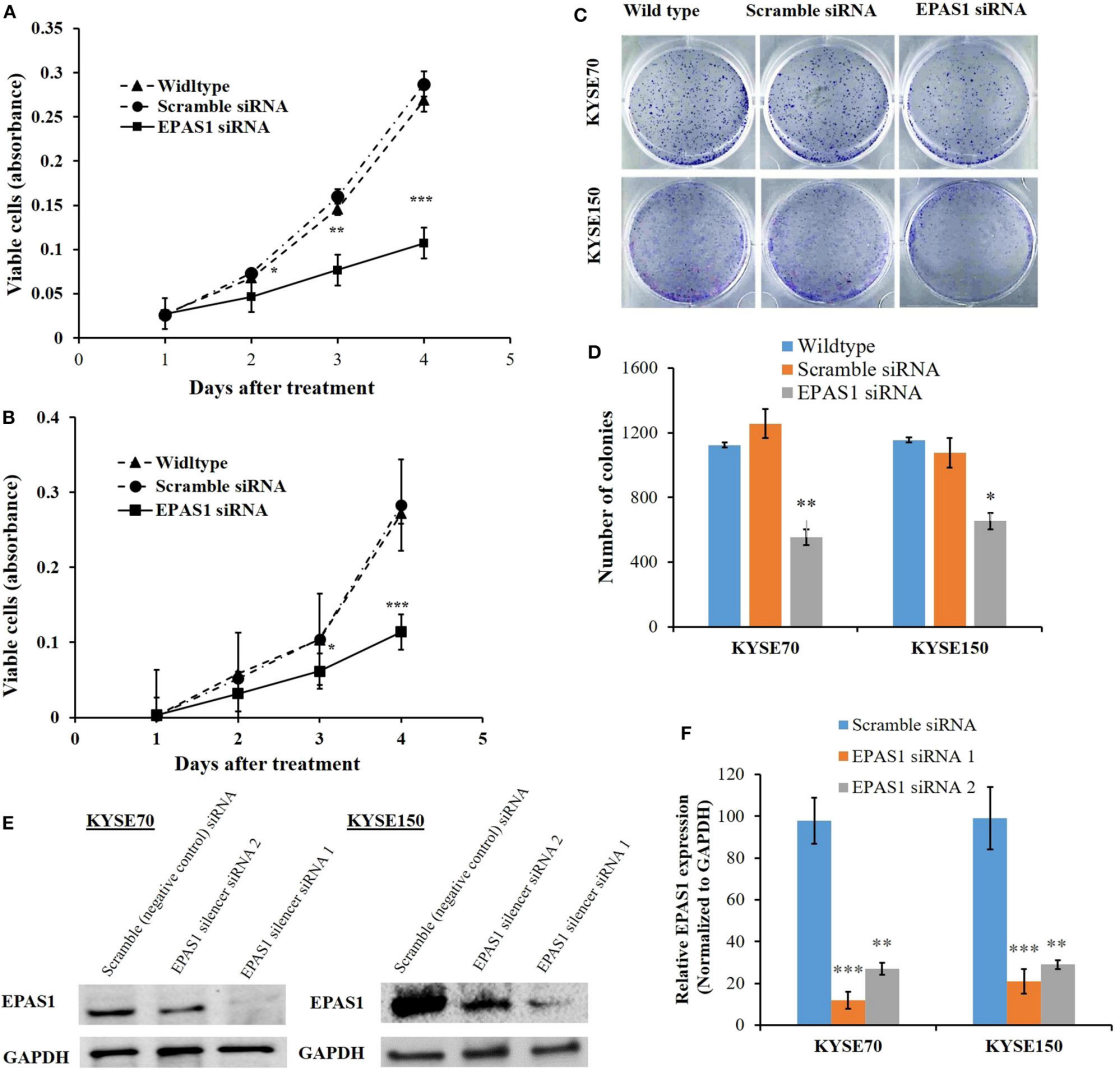
EPAS1 suppression inhibited ESCC cells proliferation and colony formation. *in vitro* suppression of EPAS1 using siRNA in KYSE70 **(A)** and KYSE150 **(B)** cells caused significant reduction in proliferation at different time points when compared with untreated and scramble control cell groups. In addition, silencing of EPAS1 induced significant reduction of colony formation capacity KYSE70 **(C)** and KYSE150 **(D)** cells in comparison to that of control groups. **(E)** Expression of EPAS1 protein in KYSE70 and KYSE150 cells followed by siRNA treatment. **(F)** Relative expression of EPAS1 in KYSE70 and KYSE150 cells followed. EPAS1 siRNA1 ans siRNA2 significantly inhibited the expression of EPAS1 in KYSE70 and KYSE150 cells. Results are shown as mean ± SD and significance level was taken at *p* < 0.05. *p < 0.05, **p < 0.01, and ***p < 0.001.

The effects of EPAS1 manipulation on ESCC cell proliferation, invasion, and migration were examined followed by *EPAS1* silencing using *EPAS1* siRNA. For cell proliferation, viable cells from KYSE70^−EPAS1^, KYSE150^−EPAS1^, KYSE70^+Scr^, KYSE150^+Scr^, KYSE70^wildtype^, and KYSE150^wildtype^ cell groups were measured on days 0–3. EPAS1 suppressive cells, KYSE70^−EPAS1^ and KYSE150^−EPAS1^, showed a significant decrease in cell proliferation when compared with control groups (KYSE70^+Scr^, KYSE150^+Scr^, KYSE70^wildtype^, and KYSE150^wildtype^), respectively (Figures 6A,B). For example, significant [46.50% (*p* < 0.05), 49.78% (*p* < 0.01), and 53.41% (*p* < 0.001)] inhibitions of KYSE70^−EPAS1^ cells proliferation were noted on days 1, 2, and 3, respectively, in comparison to that of KYSE70^+Scr^ cells (Figure 6A). Similar results were noted in the case of KYSE150^−EPAS1^, exhibiting 39.06%, 40.99% (*p* < 0.05), and 59.72% (*p* < 0.001) inhibition on days 1, 2, and 3, respectively, in comparison to that of KYSE150^+Scr^ cells (Figure 6B).

Silencing of EPAS1 caused a significant reduction of clonogenic capacity of ESCC cells (KYSE70^−EPAS1^ and KYSE150^−EPAS1^) in comparison to the controls (KYSE70^+Scr^ and KYSE150^+Scr^) and nontransfected wild-type (KYSE70^wildtype^ and KYSE150^wildtype^) ESCC cells (Figures 6C,D). A 55.85% reduction of colony formation in KYSE70^−EPAS1^ was observed in comparison to the control KYSE70^+Scr^ cells (Figure 6C; *p* < 0.01). Similarly, 43.32% reduction in colony formation capacity was noted by the KYSE150^−EPAS1^ cells when compared to that of KYSE150^+Scr^ control cells (Figure 6D; *p* < 0.05).

### Silencing of EPAS1 Reduced Wound Healing, Migration, and Invasion of ESCC Cells

**Figure 7:**
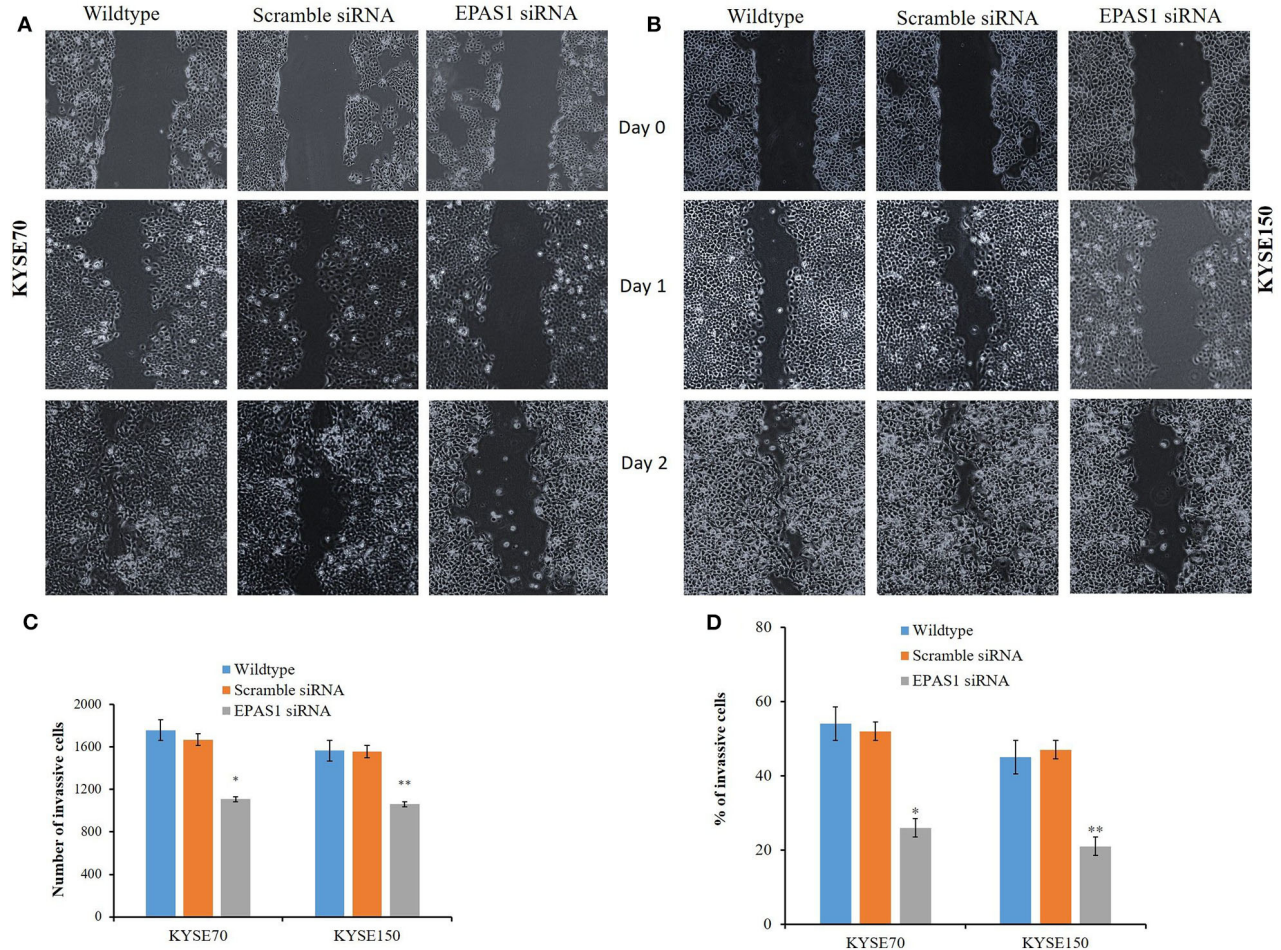
Silencing of EPAS1 causes reduction of wound healing, invasion and migration of ESCC cells. Silencing of EPAS1 causes inhibition of wound healing of ESCC cells as suppression of EPAS1 induced reduction of migration capacity of KYSE70 **(A)** and KYSE150 **(B)** cells, thereby healing the wound more slowly in comparison to that of untreated wild type and scramble control cells. Similarly, a significantly reduced population of KYSE70 **(C)** and KYSE150 **(D)** cells exhibited invasion and migration followed by suppression of *EPAS1* when compared to that of untreated or scramble control cells. Results are shown as mean ± SD, and significance level was taken at *p* < 0.05. *p < 0.05 and **p < 0.01.

The ESCC cells with reduced EPAS1 expression (KYSE70^−EPAS1^ and KYSE150^−EPAS1^) cells showed significant (*p* < 0.01) reduction in wound healing, invasion, and migration capacity when compared with the control and nontransfected wild-type cancer cells (Figure 7). KYSE70^−EPAS1^ and KYSE150^−EPAS1^ ESCC cells had lower cell migration potential than the controls (KYSE70^+Scr^ and KYSE150^+Scr^) and wild-type (KYSE70^wildtype^ and KYSE150^wildtype^) cells as they healed the created scratch slowly when compared to their counterpart (Figures 7A,B). KYSE70^−EPAS1^ and KYSE150^−EPAS1^ cells took more time in healing the wounds, whereas nontreated and control cells took less time to heal the wounds. Similarly, KYSE70^−EPAS1^ and KYSE150^−EPAS1^ had reduced barrier penetration and migration potential in BME-coated invasion chamber when compared with control and nontreated cancer cells (Figures 7C,D). The relative fluorescence unit (which is proportional to the BME-barrier invading cells) from KYSE70^−EPAS1^ and KYSE150^−EPAS1^ cells was significantly less in comparison to that of KYSE70^+Scr^ and KYSE150^+Scr^ and KYSE70^wildtype^ and KYSE150^wildtype^ cells. KYSE70^−EPAS1^ cells showed 50% reduction of invasion and migration when compared to that of KYSE70^+Scr^ cells (Figure 7C; *p* < 0.05), whereas KYSE150^−EPAS1^ cells exhibited 55.32% reduction of invasion and migration in comparison to that of KYSE150^+Scr^ cells (Figure 7D; *p* < 0.01).

## Discussion

This study reported the molecular dysregulation, its clinical significance, and functional insights of *EPAS1* in the pathogenesis of ESCC. The results implied that *EPAS1* plays an important role in carcinogenesis of ESCC through regulation of cellular proliferation, migration, and invasion and thus acts as an oncogene.

Mutations of *EPAS1* has been identified in various cancers such as in paraganglioma (21), pheochromocytoma (12), and pancreatic carcinomas (22). In addition, data analysis from the International Cancer Genome Consortium (ICGC) revealed that mutations in *EPAS1* are common in many human malignancies, including esophageal cancer (adenocarcinoma) (https://dcc.icgc.org/). It was shown that 23.72% (*n* = 97/409) of patients with esophageal adenocarcinoma had somatic mutations in *EPAS1*. However, there are no data available regarding the mutational status of *EPAS1* in ESCC in the ICGC database. In the present study, we have detected *EPAS1* mutations in 7.5% (*n* = 6/80) patients with ESCC. The computational analysis revealed that the variants identified in the current study are novel and could have the potential to affect the functionality of the protein. The four frameshift variants (V382Gfs^*^12, A381Lfs^*^13, K379Ifs^*^6, and K364Nfs^*^12) may cause NMD, resulting in strongly truncated nonfunctional protein production. However, further functional studies with these variants are needed to confirm their roles in generating NMD or truncated protein product. The other variants (c.1099C>A, c.1093C>G, c.1121T>G, and c.1091A>T) may cause a change in the primary structure of the protein and may lead nonfunctional/overfunctional protein as they showed deleterious/diseases causing effects on protein in computational prediction. Therefore, further studies are warranted to validate the functional implications of the variants identified in the present study.

This is the first study reporting *EPAS1* mutations in patients with ESCC and their clinical implications. The association of *EPAS1* mutations with the presence of lymph node metastasis indicates that mutations in *EPAS1* sequence could be predictive makers for lymph node metastasis. Also, younger patients (≤60 years old) are predicted to be more likely to harbor *EPAS1* mutations. In addition, the trends of poorer survival rates (mutant = 570 days vs. nonmutant = 2,097 days) of patients with ESCC having *EPAS1* mutations could help to predict the clinical outcome of these patients. However, the difference did not reach statistical significance, maybe due to the low number (*n* = 6) of positive populations.

DNA copy number alterations and dysregulated expression of genes are common in human cancers and are being used as biomarkers of the disease (37). Dysregulation of *EPAS1* is associated with the carcinogenesis of different types of cancers such as lung carcinoma (8), renal cell carcinoma (9), hepatocellular carcinoma (10), neuroblastoma (11), pheochromocytoma (12), glioma (13), and colorectal adenocarcinoma (14). Tumor-promoting oncogenic roles of *EPAS1* was noted in the pathogenesis of lung carcinoma, renal cell carcinoma, liver neuroblastoma, pheochromocytoma, and so on (8–12), whereas other studies reported the tumor-suppressive properties in the pathogenesis of glioma, colorectal carcinoma, and neuroblastoma (13, 14, 38). For example, EPAS1 expression is associated with a better outcome of patients with neuroblastoma and low-risk tumors (38). In this study, amplification or deletion of *EPAS1* DNA number (87.5%; *n* = 70/80) followed by mRNA higher or lower expression (73.7%; *n* = 59/80) in tissue samples indicates its regulatory roles in progression of ESCC. Several studies also noted higher or lowered expression of *EPAS1* both in mRNA and protein levels in patients with other cancers (14–16, 39). The present study for the first time reported the deregulation of *EPAS1* in ESCCs, which are in consistence with other studies.

The association of *EPAS1* DNA number amplification or deletion with tumor site and tumor stages indicated the heterogeneous nature of ESCC. The biological aggressiveness, surgical accessibility, and molecular makeup of ESCC from different sites of the esophagus, upper site (proximal), and the middle/lower site (distal) are different (40). Thus, it is not surprising that *EPAS1* DNA number is different in these two portions of the esophagus. In addition, the genetic and epigenetic makeup of different tumor stages is different (40). Thus, ESCC of different T stages showed a different level of *EPAS1* DNA number in the present study. Finally, the poorer survival rates of patients with stage III ESCC having *EPAS1* DNA amplification implied the prognostic significance of *EPAS1* in ESCC (Figure 4C). Therefore, *EPAS1* DNA changes could have the potential to be used as a prognostic marker for patients with ESCC.

DNA copy number aberrations are frequent acquired changes in cancers, which lead to abnormal expression of genes and play crucial roles in pathogenesis and progression of ESCC (40, 41). The correlation of *EPAS1* DNA number amplification and increased mRNA expression in ESCC in the present study indicated that hypoxic tumor niche induces alterations of *EPAS1*, which in turn can promote carcinogenesis. Furthermore, DNA amplification and higher mRNA expression in ESCC harboring mutations indicated the concerted aberration of *EPAS1* in ESCC. Thus, the molecular dysregulation of *EPAS1* detected in the present study could stimulate carcinogenesis.

The functional roles of *EPAS1* in ESCC have been studied, followed by siRNA-mediated silencing in ESCC cells. A significant reduction of cancer cell proliferation and colony formation capacity in comparison to that of untreated wild-type and scramble control groups were noted (Figure 6). The findings of the present study are in concurrence with previous reports on various types of cancers, including clear cell renal cell carcinoma, pancreatic adenocarcinoma, and breast carcinoma (9, 42, 43). Silencing of *EPAS1* via siRNA induced reduced cell proliferation, increased apoptosis, and generated smaller tumor in a mouse model of pancreatic carcinoma (43), whereas inhibition of EPAS1 with a small molecular target (PT2399) causes tumor regression in a preclinical mouse model of primary and metastatic clear cell renal cell carcinoma (9). Our results and available information in the literature implied that *EPAS1* could be a potential target for developing effective therapeutics for better management of patients with cancer. However, some other studies reported tumor inhibitory functionality of EPAS1 in various cancer models (38, 44). For example, treatment with EPAS1 inhibitors did not block *in vitro* neuroblastoma cell proliferation or xenograft growth in the mouse model (38). Furthermore, HIF-2α inhibited *in vivo* growth of cells from high-grade soft tissue sarcomas. Loss of HIF-2α promoted proliferation of sarcoma and increased calcium and mTORC1 signaling in undifferentiated pleomorphic sarcoma and dedifferentiated liposarcoma (44).

EPAS1 promotes angiogenesis in mouse models by inducing both vascular endothelial growth factor and its receptor Fms related tyrosine kinase 1 expression in endothelial cells (45). Furthermore, suppression of *EPAS1* using shRNA in breast carcinoma cells reduced the cellular growth and inhibited angiogenesis (42). Inconsistent with the previous study, we noted that silencing of *EPAS1* inhibited the wound healing and migration capacity when compared to that of untreated and scramble control groups of ESCC cells. Similarly, suppression of *EPAS1* showed a significant reduction in barrier penetration and invasion, indicating its lower metastatic potential in comparison to that of control ESCC cells. Thus, the therapeutic strategies targeting EPAS1 could have the potential for effective inhibition of cancer cell growth, migration, and invasion.

To conclude, the present study for the first time detected multiple novel *EPAS1* mutations in ESCC. These mutations may contribute to the altered expression and/or structural and functional changes of the gene, which in turn could play an essential role in the pathogenesis of the disease. In addition, the association of molecular dysregulation in DNA number, mRNA expression, and mutations in ESCC along the clinical significance of the gene has provided critical insights of tumor-promoting properties of EPAS1 in the pathogenesis of ESCC. Therefore, the results of this study will enrich the current understanding of *EPAS1* in directing carcinogenesis of ESCC, as well as opening new opportunities for the development of novel therapeutic strategies against cancer.

## Data Availability Statement

The raw data supporting the conclusions of this article will be made available by the authors, without undue reservation.

## Ethics Statement

The studies involving human participants were reviewed and approved by Ethical approval for this work has been obtained from the Griffith University Human Research Ethics Committee (MED/19/08/HREC). The patients/participants provided their written informed consent to participate in this study.

## Author Contributions

FI carried out most of the experiments and draft the manuscript. VG plan the project and revised the manuscript. SL manage the clinical data. AL analyze the clinical data and revised the manuscript. SP supervise and collect the funding for the project. All authors contributed to the article and approved the submitted version.

## Conflict of Interest

The authors declare that the research was conducted in the absence of any commercial or financial relationships that could be construed as a potential conflict of interest.
